# Predictors of Metabolic Syndrome in Participants of a Cardiac Rehabilitation Program

**DOI:** 10.5402/2012/736314

**Published:** 2012-03-28

**Authors:** Alejandra Farias Godoy, Andrew Ignaszewski, Jiri Frohlich, Scott A. Lear

**Affiliations:** ^1^Department of Biomedical Physiology and Kinesiology, Simon Fraser University, Burnaby, BC, Canada V5A 156; ^2^Healthy Heart Program, St. Paul's Hospital, Providence Health Care, Vancouver, BC, Canada V6Z 1Y6; ^3^Faculty of Health Sciences, Simon Fraser University, Harbour Centre Campus, Vancouver, BC, Canada V6B 5K3

## Abstract

Metabolic syndrome increases the risk of all-cause mortality, cardiovascular mortality and cardiovascular events in patients with cardiovascular disease (CVD). This study assessed the predictors of metabolic syndrome, both its incidence and resolution in a cohort of cardiac rehabilitation program graduates. *Methods*. A total of 154 and 80 participants without and with metabolic syndrome respectively were followed for 48 months. Anthropometric measurements, metabolic risk factors, and quality of life were assessed at baseline and at 48 months. Logistic regression models were used to assess the predictors of metabolic syndrome onset and resolution. *Results*. Increasing waist circumference (OR 1.175, *P* ≤ 0.001) was an independent predictor for incident metabolic syndrome (*R* ^2^ for model = 0.46). Increasing waist circumference (OR 1.234, *P* ≤ 0.001), decreasing HDL-C (OR 0.027, *P* = 0.005), and increasing triglycerides (OR 3.005, *P* = 0.003) were predictors of metabolic syndrome resolution. *Conclusion*. Patients with CVD that further develop metabolic syndrome are particularly susceptible for the cascade of cardiovascular events and mortality. Increasing waist circumference confers a higher risk for future onset of metabolic syndrome in this group of patients. They will require closer follow-up and should be targeted for further prevention strategies after cardiac rehabilitation program completion.

## 1. Introduction

The metabolic syndrome (MetS) is a prothrombotic, proinflammatory state characterized by abdominal obesity, insulin resistance/hyperinsulinemia (leading to elevated plasma glucose), elevated blood pressure (BP) and dyslipidemia (high triglycerides, low-high-density lipoprotein [HDL-C] with preponderance of small-dense, low-density lipoprotein [LDL-C] particles) [1]. These metabolic changes are associated with the development and progression of diabetes and coronary artery disease [2, 3]. Previous studies have reported that for patients with cardiovascular disease (CVD), MetS confers an even greater risk for cardiovascular events, including cardiovascular and all-cause mortality than those without MetS [4–7]. For example, MetS risk estimates for all-cause and cardiovascular mortality were reported to be greatest in a population with existing CVD compared to those without CVD (HR 1.47 versus 1.06, HR 2.53 versus 2.01, *P* < 0.05, resp.) [5]. Likewise, the presence of MetS in women with suspected coronary artery disease confers a significantly higher risk for cardiovascular events than in those with normal metabolic status (HR 4.93 versus 1.41, *P* = 0.05) [6]. As there is a synergistic effect between MetS and CVD on cardiovascular morbidity and mortality [7], these patients are at especially high risk, and it is therefore important to identify MetS in patients with CVD as well as find the best predictors of it.

Cardiac rehabilitation programs (CRPs) are uniquely positioned to assess the presence of MetS and are a proven intervention in reducing CVD risk [8–10]. The purpose of this study was to identify predictors of MetS in a cohort of men and women with CVD free of MetS, over a four-year period following completion of their CRP. We also assessed predictors of MetS resolution in men and women with both CVD and MetS over the same time period. Specific attention was placed on identifying predictors that can be easily measured outside of the CRP environment and readily available at the health care provider's office without substantial additional cost.

## 2. Methods

We used data from the Extensive Lifestyle Intervention (ELMI) Trial in which a total of 302 men and women with ischemic heart disease were recruited following completion of a standard CRP for a four-year study [11]. The ELMI Trial was a randomized study to test the effectiveness of a modest intervention of additional exercise sessions, telephone follow-up, and risk factor and lifestyle counselling compared to usual care. For the current analysis, the two groups have been combined and analyzed together as there were no differences between the two groups in outcomes relevant to the current investigation.

The MetS was defined according to the most recently published harmonized criteria by the International Diabetes Federation in conjunction with the National Heart, Lung, and Blood Institute, American Heart Association, World Heart Federation, International Atherosclerosis Society, and International Association for the Study of Obesity [12]. Participants were classified as having MetS at CRP completion and at 48 months if they had three out of the following five criteria: triglycerides ≥ 1.7 mmol/L, HDL-C < 1.00 mmol/L for men and <1.30 mmol/L for women or specific treatment for these lipid abnormalities, BP ≥ 130/85 mmHg or specific treatment of diagnosed hypertension, fasting plasma glucose ≥ 5.6 mmol/L, or previously diagnosed diabetes [12]. The majority of participants in the ELMI study were Caucasian; therefore, we chose the waist circumference cut points for Europeans of ≥94 cm for men, ≥80 cm for women. The cohort was retrospectively assigned into the following categories at CRP completion: (1) “New onset MetS” (participants who had no MetS at baseline but had MetS at 48 months), (2) “MetS resolution” (participants who had MetS at baseline but did not have MetS at 48 months), (3) “Always MetS” (participants who had MetS at baseline and 48 months), and (4) “Never MetS” (participants who had no MetS at baseline and 48 months).

All participants underwent a baseline and a 48-month assessment that consisted of cardiovascular risk factors (lipid profile, fasting blood glucose, and BP), lifestyle (exercise capacity, leisurely exercise, and anthropometric measurements) and psychosocial parameters (perceived stress, illness intrusiveness, health-specific, and exercise self-efficacy). Body mass index (BMI) was calculated from height (in metres) and weight (in kg) with participants in street clothing and shoes removed. Waist circumference was measured in cm at the point of maximal narrowing of the trunk viewed from the anterior position with the participant standing upright following a normal expiration [13]. Systolic and diastolic BP were measured in mmHg and assessed with a manual sphygmomanometer, recorded as the average of two measures taken two minutes apart after five minutes of seated rest. Fasting lipid profile (total cholesterol (TC), HDL-C, and triglycerides) and fasting blood glucose were assessed using standard laboratory methodology [14]. Calculation of LDL-C was done using the Friedwald equation [15]. Exercise capacity was determined by a symptom-limited stress test and expressed in metabolic equivalents (METS). Health-specific self-efficacy was used to assess the participant's confidence to achieve successful lifestyle changes and was measured by a questionnaire based on the Likert scoring [16]. Perceived stress and illness intrusiveness were assessed through the Perceived Scale Rating [17] and the Illness Intrusive Rating [18] based on the Likert scoring. These questionnaires evaluate an individual's perception of stress in different situations and the perception of how their illness burden affects different aspects of their lives, respectively.

## 3. Statistical Analysis

Baseline comparisons between the groups were done with one-way ANOVA and Pearson's chi square for continuous and categorical variables, respectively. Multiple comparisons were assessed with the Tukey correction for variables that reached significance. Paired sample *t*-tests were used to compare risk factors at baseline and at 48 months within each group. Multinomial logistic regression models were used to assess for independent predictors of: (1) “New onset MetS”, and (2) “MetS resolution.” Age and sex were entered a priori. Variables were considered in the equation according to their association with the MetS; therefore, exercise variables such as baseline values of fitness level in metabolic equivalents, TC, LDL-C, triglycerides, HDL-C, glucose, waist circumference and BP as well as psychosocial parameters such as illness intrusiveness, perceived stress, and health-specific self-efficacy were considered. Group assignment (intervention group versus usual care group), was also entered as a variable in the equations; however, as it was not a significant contributor to our outcomes of interest, it was not included in the final models. Medication intake at baseline for statins, ACE inhibitors, calcium channel blockers, diabetes-related medications, and beta blockers (taking the medication versus not taking the medication) were also entered as variables in the equations; however, as they were not predictive of our outcomes, they were not included in the final models. Variables were removed in a backwards fashion according to their correlation to other variables in the equation and their predictive value at each step. As part of the model, special attention was placed on those variables that can be easily assessed in the health care provider's office such as anthropometric measurements, BP and psychosocial parameters such as illness intrusiveness, perceived stress, and health-specific self-efficacy. These variables were entered in the equation with age and sex, without variables that needed laboratory measurements. This was done to address our purpose to identify predictors that can be measured in the health care provider's office. These models were based on 234 participants who had complete data. The *P* value was set at 0.05. All data were analyzed using SPSS version 18.

## 4. Results

Of the 302 participants, 56 did not have outcome data at 48 months, and 12 had incomplete assessments. Those that were lost to follow-up or had an incomplete assessment were not significantly different from the participants included in the present analysis (data not shown). This resulted in a total of 234 participants who were retrospectively assigned as follows: 121, 52, 28, and 33 participants in the “Never MetS,” “Always MetS,” “MetS resolution” and “New onset MetS” groups, respectively.  Table 1 shows baseline comparisons of demographic and cardiovascular risk factors between the groups. There were no significant differences in age, sex, family history of CVD, coronary artery bypass graft or percutaneous transluminal angiography, history of myocardial infarction, TC, LDL-C, diastolic BP, exercise capacity, leisure time physical activity, perceived stress, health-specific and exercise self-efficacy among the four groups. The “Never MetS” group had a lower proportion of participants with diabetes, lower waist circumference and BMI than the other groups, as well as higher HDL-C and lower triglycerides than the “Always MetS” and “MetS resolution” groups. The “Never MetS” group had lower fasting blood glucose than the “Always MetS” group and lower illness intrusiveness than the “MetS resolution” group. The “New onset MetS” group had a lower proportion of participants with diabetes and lower triglycerides than the “Always MetS” and “MetS resolution” group, as well as higher HDL-C, lower BMI and lower waist circumference than the “Always MetS” group. The “Always MetS” group had a higher systolic BP than the “New onset MetS” group.

Table 2 shows CVD risk factors at baseline and at 48 months for the four groups. The “Never MetS” group had an increase in HDL-C, diastolic BP and waist circumference, as well as a decrease in triglycerides, illness intrusiveness, exercise capacity, and self-reported leisure time physical activity (*P* ≤ 0.05). The “Always MetS” group had a decrease in TC, perceived stress, exercise capacity, self-reported leisure time physical activity, and exercise self-efficacy (*P* ≤ 0.05) as well as an increase in waist circumference (*P* ≤ 0.05). The “MetS resolution” group had a decrease in TC, triglycerides and self-reported leisure time physical activity (*P* ≤ 0.01) as well as an increase in HDL-C and exercise capacity (*P* ≤ 0.05). The “New onset MetS” group had a decrease in HDL-C, exercise capacity and exercise self-efficacy an increase in triglycerides, systolic and diastolic BP, and BMI and waist circumference (*P* ≤ 0.05).

Table 3 outlines the results of the multinomial logistic regression models to identify predictors for MetS onset at 48 months and MetS resolution at 48 months. All possible baseline parameters were considered; waist circumference was a significant predictor of MetS onset (*P* ≤ 0.001, *R* ^2^ = 0.46) after adjusting for age and sex. That is, an increase in waist circumference was associated with an increase in the risk of MetS. Model 2 shows the result of the logistic regression model for independent predictors of MetS resolution at 48 months. Baseline waist circumference, HDL-C and triglycerides were independent predictors of MetS resolution after adjusting for age and sex (*P* ≤ 0.01, *R* ^2^ = 0.59). This indicates that an increase in waist circumference as well as an increase in triglycerides was associated with a greater possibility of MetS resolution, while a decrease in HDL-C was associated with a greater possibility of MetS resolution.

## 5. Discussion

Our data show that increasing waist circumference was the only significant independent predictor of MetS after adjustment for age and sex. Paradoxically, waist circumference was also an independent predictor of MetS resolution as well as decreasing HDL-C and increasing triglycerides after adjustment for age and sex. An interesting finding was that those participants that did not change their metabolic status, the “Never MetS” group and the “Always MetS” group, who represent the lowest and highest risk groups respectively, experienced a deterioration of exercise capacity and waist circumference during a four-year follow-up after CRP completion.

Waist circumference has a strong association with the metabolic syndrome cluster of abnormalities which in turn strongly correlate with the development of CVD [19–21]. In those with CVD, the concomitant existence of the MetS cluster of abnormalities confers an even higher risk for cardiovascular events, cardiovascular and all-cause mortality [4–7]. Therefore, predicting the incidence of MetS in individuals with CVD is important. Our findings show that CRP graduates with an enlarged waist are at increased risk of developing MetS in the next four years such that for every 5 cm increase in waist circumference the risk of developing MetS in the next 48 months increased by 87.5%. Together, with age and sex, this model accounted for 46% of the variation in new onset of MetS. Given that waist circumference is a component of the MetS, it is not unexpected that an enlarged waist would predict future onset of MetS. However, it is important to note that none of the other four components (triglycerides, HDL-C, fasting glucose, or BP) were predictive.

The relation between waist circumference and the rest of the parameters of the metabolic syndrome is well documented; increasing values of waist circumference have been associated with increasing triglyceride levels, fasting insulin and glucose levels as well as decreasing HDL-C levels in healthy middle-aged men and women [22]. Fox et al. reported that waist circumference is highly correlated with visceral adipose tissue in both men and women (*r* = 0.73 and 0.78, *P* ≤ 0.001, resp.) and, in turn, visceral adipose tissue is highly correlated with HDL-C, systolic BP, diastolic BP, and blood glucose [19]. Surprisingly, although the measurement of waist in the clinical setting has increased in the past 10 years, it is still under used in clinical practice [23]. Identifying the risk for developing MetS amongst high-risk patients is the next step to restratify this risk group. Increased waist circumference was also positively associated with a greater resolution of MetS in addition to lower HDL-C and higher triglycerides levels at baseline. This might be explained in part by the patient's higher drive to improve their risk factor profile by a perception of a higher degree of disease burden. However, given that we did not observe improvements in anthropometric measurements, exercise capacity or psychosocial variables, which would be reflective of changes in lifestyle, it is possible that the higher-risk factor levels may have resulted in the patients being more readily identified for pharmacological treatment, with a higher proportion of high-risk patients being on lipid-lowering medications (data not shown). This is reflected by the finding that the “MetS resolution” group experienced improvements of TC, HDL-C, and triglycerides without any improvement in waist circumference. It is important to mention that having an enlarged waist at CRP completion and a decreased waist at 48 months is what defined the “MetS resolution” group; therefore, it is not surprising to find that waist circumference was associated with MetS resolution. The fact that an increased waist circumference was also associated with MetS onset over 48 months indicates the importance of managing these patients effectively whether through lifestyle changes orpharmaco therapy to reduce their future risk of CVD.

While not directly associated with our primary question, an interesting finding was that patients in either the “Never MetS” or “Always MetS” had a decrease in exercise capacity and an increase in waist circumference. This is comparable to the general CRP population, where it has been reported that adherence to favourable lifestyle behaviours decrease shortly after CRP completion [24]. This may reflect the challenges that both, the patients and the physicians have to successfully manage cardiovascular risk factors through behavioural changes [25–27]. Lifestyle changes are essential in the management and prevention of the MetS [28], and associated complications such as diabetes [29] and cardiovascular events [30]. The “New onset MetS” group had an increase in BP, anthropometric measurements and triglycerides and a decrease in HDL-C, exercise capacity and exercise self-efficacy. Furthermore, the highest risk group, “Always MetS,” as well as the lowest risk group, “Never MetS,” experienced both an increase in waist circumference and a decrease in exercise capacity, which in turn reflects deterioration of lifestyle behaviours. The importance of maintaining healthy lifestyle behaviours is vastly documented. Myers et al. reported that an improvement of 1 MET in exercise capacity (defined as 3.5 mL O2/Kg/min) provides a 12% reduction in all-cause mortality in healthy men [30], and an 18% reduction in all-cause mortality in men with diabetes [31]. Our results highlight the failure of most patients to maintain positive behavioural changes, even after a CRP. This is an important caveat that needs to be addressed in order to prevent the appearance of MetS in patients with CVD and in the long-term management of patients after CRP.

## 6. Study Limitations

As the groups were retrospectively assigned, we cannot exclude the possibility of a bias in the group assignment. Also the ELMI Trial was not designed to prospectively assess incident MetS. However, our findings, supported by the results of previous studies do suggest that the assessment of waist circumference in a high-risk population can help physicians identify those at greatest risk. We must also acknowledge that our study population, namely, patients completing a CRP, may not be representative of the general population of CVD patients. However, we have no reason to believe that waist circumference would be any less important in patients not attending a CRP. Lastly, we assessed the predictors of MetS resolution and incidence in a cohort primarily of European descent, and our results may not be applicable to other ethnic groups.

## 7. Conclusion

Over a four-year follow-up period, an increasing waist circumference is the only independent predictor of future MetS in patients with CVD. Likewise, an increasing waist was also a predictor of MetS resolution, as were increasing triglyceride levels and decreasing HDL-C. These findings strengthen the notion that patients with CVD with an enlarged waist at CRP completion are at risk of further cardiometabolic deterioration. Our results highlight the clinical importance of waist circumference for identifying and restratifying high-risk patients. Waist measurement is readily available to identify individuals at the highest risk—those with CVD at risk of future MetS. A cautionary note is that patients who did not have MetS at baseline or at 48 months still experienced an increase in waist circumference and a decrease in exercise capacity. This also occurred in patients with the MetS at baseline who still met the criteria at 48 months, indicating that both the lowest and highest risk patients are susceptible to those adverse changes and need to be regularly assessed and appropriately managed. Traditionally, the MetS has been used to assess the risk of future CVD and diabetes, whereas our study has the strength of considering the MetS and its synergistic effect with CVD as an endpoint that confers high cardiometabolic risk, and therefore, should be prevented. Our data show that waist circumference is a predictor of both, MetS onset and resolution; therefore, those patients with CVD and an enlarged waist need further risk factor management and follow-up. These results provide further data on patients with CVD at risk of MetS. An extension of CRP and/or acloser follow-up after CRP completion for patients with CVD and an enlarged waist would be a practical intervention that may improve quality of care.

## Figures and Tables

**Table 1:** Baseline comparison of demographics and cardiovascular risk factors between “Never MetS”, “Always MetS”, “MetS new onset” and “MetS resolution” groups.

Risk factors at baseline	Mean and SD and counts and percentages				Overall *P* value	Multiple comparisons
	Never MetS (*n* = 121)	Always MetS (*n* = 52)	MetS resolution (*n* = 28)	New onset MetS (*n* = 33)		
Age (years)	66.2 ± 10.3	64.5 ± 8.3	65.3 ± 7.4	62.9 ± 7.7	0.295	—
Sex (male)	98 (81%)	41 (79%)	24 (86%)	29 (88%)	0.689	—
Family history of CVD	40 (33%)	16 (31%)	7 (25%)	15 (45%)	0.360	—
Coronary artery bypass surgery	45 (37%)	20 (38%)	8 (29%)	10 (30%)	0.723	—
Percutaneous transluminal coronary angiogram	47 (39%)	14 (27%)	12 (43%)	13 (39%)	0.401	—
Diabetes	8 (6%)	20 (38%)	9 (32%)	7 (21%)	<0.001	<0.001
						(0 ≠ 1,2, 3)
						(3 ≠ 1,2)
Previous myocardial infarction	62 (51%)	24 (46%)	12 (43%)	23 (69%)	0.121	—
TC (mmol/L)	4.38 ± 0.89	4.66 ± 0.99	4.43 ± 0.73	4.48 ± 0.74	0.282	—
LDL-C (mmol/ L)	2.53 ± 0.73	2.59 ± 0.81	2.44 ± 0.58	2.68 ± 0.62	0.582	—
HDL-C (mmol/ L)	1.25 ± 0.31	0.99 ± 0.22	1.01 ± 0.20	1.17 ± 0.28	<0.001	<0.001 (0 ≠ 1,2)
						0.024 (1 ≠ 3)
Triglycerides (mmol/ L)	1.32 ± 0.66	2.37 ± 0.84	2.15 ± 1.11	1.38 ± 0.50	<0.001	<0.001 (0 ≠ 1,2)
						<0.001 (3 ≠ 1,2)
Fasting blood glucose (mmol/L)	5.32 ± 1.58	6.34 ± 1.41	5.99 ± 1.18	5.86 ± 1.30	<0.001	<0.001 (0 ≠ 1)
Systolic BP (mmHg)	126 ± 22	134 ± 17	130 ± 27	120 ± 14	0.024	0.020 (1 ≠ 3)
Diastolic BP (mmHg)	71 ± 10	75 ± 10	72 ± 13	73 ± 10	0.154	—
Body mass index (kg/m²)	25.4 ± 2.9	30.4 ± 4.2	28.6 ± 3.9	27.6 ± 3.1	<0.001	<0.001
						(0 ≠ 1,2, 3)
						<0.002 (1 ≠ 3)
Waist circumference (cm)	86.7 ± 9.0	102.4 ± 11.5	99.8 ± 11.0	95.6 ± 9.1	<0.001	<0.001
						(0 ≠ 1,2, 3)
						0.013 (1 ≠ 3)
Exercise capacity (METS)	10.8 ± 4.4	9.1 ± 2.2	13.8 ± 2.9	10.7 ± 2.3	0.109	—
Leisure time physical activity (kcal/week)	3029 ± 337	2837 ± 1708	3090 ± 558	3219 ± 226	0.894	—
Perceived stress	32 ± 8	31 ± 8	34 ± 9	32 ± 6	0.512	—
Illness intrusiveness	27 ± 11	33 ± 16	36 ± 19	28 ± 13	0.020	0.040 (0 ≠ 2)
Health-specific self-efficacy	43 ± 4	42 ± 4	42 ± 6	41 ± 4	0.064	—
Exercise self-efficacy	67 ± 12	70 ± 10	63 ± 13	64 ± 12	0.080	—

CVD: cardiovascular disease, TC: total cholesterol, LDL-C: low density lipoprotein cholesterol, HDL-C: high-density lipoprotein cholesterol.

One-way ANOVA and Pearson's chi square for continuous and categorical variables, respectively. Multiple comparisons were assessed with the Tukey correction. Multiple comparisons: 0: Never MetS, 1: Always MetS, 2: MetS resolution, 3: New onset MetS.

Note: 0 ≠ 1 means that Never MetS is significantly different with Always MetS group.

**Table 2:** CVD risk factors at baseline and at 48 months for “Never MetS,” “Always MetS,” “MetS new onset” and “MetS resolution” groups.

CVD risk factors	Never MetS (*n* = 121)		Always MetS (*n* = 52)		MetS resolution (*n* = 28)		New Onset MetS (*n* = 33)	
	Baseline	48 months	Baseline	48 months	Baseline	48 months	Baseline	48 months
TC (mmol/L)	4.39 ± 0.90	4.32 ± 0.90	4.67 ± 0.99	4.40 ± 0.95^‡^	4.43 ± 0.73	4.07 ± 0.77^‡^	4.51 ± 0.74	4.55 ± 0.83
LDL-C (mmol/L)	2.52 ± 0.74	2.48 ± 0.76	2.57 ± 0.80	2.41 ± 0.69	2.45 ± 0.59	2.29 ± 0.66	2.71 ± 0.62	2.64 ± 0.71
HDL-C (mmol/L)	1.24 ± 0.30	1.30 ± 0.36^†^	0.99 ± 0.22	0.98 ± 0.21	1.01 ± 0.20	1.18 ± 0.26^†^	1.17 ± 0.28	1.09 ± 0.27^§^
Triglycerides (mmol/L)	1.32 ± 0.67	1.18 ± 0.60^‡^	2.37 ± 0.85	2.34 ± 2.20	2.16 ± 1.11	1.30 ± 0.50*	1.38 ± 0.51	1.79 ± 0.84^†^
Fasting blood glucose (mmol/L)	5.3 ± 1.6	5.3 ± 0.8	6.33 ± 1.42	6.67 ± 1.47	5.9 ± 1.2	5.6 ± 0.7	5.7 ± 1.2	6.2 ± 1.4
Systolic BP (mmHg)	126 ± 21	127 ± 21	134 ± 17	134 ± 17	130 ± 27	122 ± 17	120 ± 14	131 ± 14^†^
Diastolic BP (mmHg)	71 ± 10	74 ± 12^†^	75 ± 10	77 ± 9	72 ± 13	71 ± 9	73 ± 10	79 ± 10^†^
Body mass index (kg/m²)	25.4 ± 2.9	25.6 ± 3.5	30.4 ± 4.2	30.7 ± 4.5	28.6 ± 3.8	28.3 ± 4.5	27.6 ± 3.0	29.1 ± 3.7*
Waist circumference (cm)	86.7 ± 8.9	88.6 ± 10.9*	102.4 ± 11.5	104.7 ± 12.8^‡^	99.8 ± 11.1	99.0 ± 12.2	95.6 ± 9.1	100.9 ± 10.9*
Exercise capacity (METs)	10.6 ± 2.4	10.0 ± 2.7^†^	9.5 ± 2.1	8.9 ± 2.3^†^	14.8 ± 22.5	17.4 ± 36.8	10.6 ± 2.3	10.1 ± 2.4^‡^
Leisure time physical activity (kcal/week)	2985 ± 2225	2230 ± 1828*	2773 ± 1616	2247 ± 2104^‡^	3066 ± 1538	1786 ± 1042^†^	2784 ± 19778	2198 ± 3011
Perceived stress	32 ± 7	32 ± 8	32 ± 8	31 ± 8	34 ± 9	33 ± 10	33 ± 6	31 ± 5
Illness intrusiveness	27 ± 11	24 ± 11^‡^	33 ± 14	30 ± 14	35 ± 19	31 ± 16	28 ± 15	27 ± 15
Health-specific self-efficacy	43 ± 4	43 ± 4	42 ± 4	42 ± 4	42 ± 5	42 ± 4	41 ± 4	40 ± 4
Exercise self-efficacy	67 ± 12	64 ± 14	70 ± 10	63 ± 15*	65 ± 11	58 ± 16	64 ± 13	58 ± 14^‡^

*P ≤ 0.001.

^†^ *P* ≤ 0.01.

^‡^ *P* ≤ 0.05.

Paired sample *t*-tests.

Total cholesterol, LDL-C: low density lipoprotein cholesterol, HDL-C: high density lipoprotein cholesterol, and BP: blood pressure.

**Table 3:** Multinomial regression models for independent predictors of “New onset MetS” 48 months (Model 1) and for independent predictors of “MetS Resolution” (Model 2) following cardiac rehabilitation.

Variables	Model 1 (*R* ^2^ = 0.46)		*P* value
	Odds ratio	95% confidence interval	
Age (years)	0.971	0.927–1.018	0.220
Sex	0.187	0.043–0.823	0.027
Waist circumference (cm)	1.175	1.102–1.253	≤0.001

Model 2 (*R* ^2^ = 0.59)			

Age (years)	1.027	0.966–1.092	0.396
Sex	0.030	0.004–0.202	≤0.001
Waist circumference (cm)	1.234	1.145–1.330	≤0.001
HDL-C (mmol/L)	0.027	0.002–0.344	0.005
Triglycerides (mmol/L)	3.005	1.465–6.166	0.003

Multinomial logistic regression models.

## List of Abbreviations

MetS: Metabolic syndrome
CVD: Cardiovascular disease
CRP: Cardiac rehabilitation programs
BP: Blood pressure
TC: Total cholesterol
HDL-C: High density lipoprotein cholesterol
LDL-C: Low density lipoprotein cholesterol
HR: Hazard ratio
ELMI Trial: Extensive Lifestyle Management Intervention Trial
BMI: Body mass index
METS: Metabolic equivalents.
